# Firearm Deaths Impacting Older Adults

**DOI:** 10.1007/s10900-024-01441-7

**Published:** 2025-01-30

**Authors:** James H. Price, Erica Payton

**Affiliations:** 1 University of Toledo, Toledo, OH 43606 USA; 2 University of North Carolina at Greensboro, Greensboro, NC USA

**Keywords:** Suicides, Homicides, Firearms, Older adults, Years of life lost

## Abstract

Each year in the United States (U.S.) thousands of older adults die from firearm-related injuries. The purpose of this study was to characterize the similarities and differences in the three main forms of firearm mortality (homicides, suicides, and unintentional) in older adults. Using the Web-based Inquiry Statistics Query and Reporting System (WISQARS) for the year 2021 we conducted a descriptive analysis (e.g. frequencies, percents, crude rates, rank orders) by gender, race/ethnicity, age, and census region of the U.S. Older adult firearm-related homicides were most likely to occur in males (61.2%), non-Hispanic whites (61.9%), ages 65–69 (42.4%) and in the South (53.6%). Firearm-related suicides were most common in males (91.4%), non-Hispanic whites (93.5%), ages 65–69 and 70–74 (24.8 and 24.7%, respectively), and in the South (45.1%). Firearm suicides were 12 times more common than firearm homicides and 99 times more common than unintentional firearm-related deaths. Both firearm homicides and suicides decreased with age. Years of potential life lost before 80 paralleled the demographic mortality data, resulting in over 45,000 potential years of life lost in 2021. These findings underscore the need to focus primary prevention of firearm-related mortality in older adults on the role of suicides, especially in non-Hispanic white males. In addition, improving mental health care access for older adults and their social connections are essential elements of preventing firearm-related suicides.

## Introduction

The United States (U.S.) population age 65 and older numbered 56, 229,126 or 16.7% of the population in 2021 [1]. Every day 10,000^+^ adults turn 65 years of age. The number of Americans aged 65 and older is projected to be 82 million by 2050, 23% of the population [2]. The older population is becoming more racially and ethnically diverse [2]. In addition, poverty in the elderly population differs by race and ethnicity: non-Hispanic Blacks (18%), Hispanics (17%), and non-Hispanic whites (8%). As the elderly population increases, we can expect to see substantially more older adults facing more challenges to the quality and quantity of their lives. Many older adults will face one or more of the following challenges: loss of friends and family members (sources of support), cognitive decline including dementias, social isolation and loneliness, physical decline including frailty, increases in chronic diseases (e.g., arthritis, cancers, cardiovascular, diabetes, etc.), mental health issues (e.g., depression, anxiety), and diminished financial resources, just to name a few of the more common challenges. For too many these challenges will not be able to be confronted with the same vigor and vitality that was used in their younger years. Additionally, the challenges become more numerous and many of them will be chronic.

The aforementioned challenges may result in changes in personality, (e.g. easy to agitate) and perceptions, (e.g. paranoia) for some older adults, including perceptions of their environments such as no one cares about them, a lack of belonging, being a burden, and a darker and more dangerous world. Some of these challenges and perceptions may result in some older adults feeling safer and self-sufficient if they can protect themselves, a pathway to owning firearms. For most older firearm owners, their firearms were likely acquired when they were younger.

In 2017, the Pew Research Center estimated that 33% of older adults owned a firearm and another 12% lived with someone who had a firearm [3]. Another study of individuals ages 50–80 years conducted at the end of 2019 found that 26.7% reported owing one of more firearms and 24.7% of them reported they stored at least one of their firearms loaded and unlocked [4]. These older adults claimed they owned firearms, primarily for protection (69.5%), especially because of fear of criminal assault (39.4%). The Centers for Disease Control and Prevention (CDC) published an eight state study (AK, CA, MN, NC, OH, OK) regarding firearm ownership and storage in 2022 and it included older adults (65^+^years). Firearm ownership varied from 15.9 to 47.7% [5]. Approximately one-half of (58.5% to 72.5%) those who stored a firearm loaded also stored it unlocked. Loaded and unlocked firearm storage is the most dangerous way to keep firearms.

Firearm violence is often perceived to be the province of the young, especially young males. However, as firearms have become more numerous in the U.S., the firearm has grown in perspective as the insurance policy and great equalizer of the weak and vulnerable [6]. Research has found that the presence of a firearm in the house increases the risks of residents becoming victims of homicides, suicides and unintentional firearm injuries [7–9]. We focus on firearm deaths of older adults by three intentions: homicides, suicides, and unintentional firearm deaths. The CDC reported on homicides in individuals 60 years of age and older during the years 2002–2016 using the National Vital Statistics System (NVSS) [10]. Almost half (46%) the homicides were perpetrated by a spouse/intimate partner, child or other relative or friend. Firearms were the weapons most commonly used (42.2%). Men were twice as likely as women to be victims and younger ages (60–69) of males were over three times as likely to be victims compared to older males (80^+^years). An international review of elder homicides conducted in 2019 found the victims were usually males, younger (60–74 years), white, the victims knew their perpetrators, (usually family members), the perpetrators used firearms (42%), had psychiatric/substance abuse problems, were part of a property crime or “heated argument”, and were committed in the victims homes [11]. A third study using the CDC WISQARS database and the National Violent Death Reporting System (NVDRS) for the years 2003–2017 characterized homicides of older adults (60 years or older) [12]. They found firearms were the most common method (44%) used to commit homicides, usually handguns. The majority of victims were males (63%), white (66%), ages 60–74 (72%), and were killed in their homes (66%). The perpetrators of the firearm homicides were males (89%), family members or ex-spouses (56%), younger than the victims (61%) and white (51%).

In contrast to firearm homicides, firearm suicides are more common [13]. A review of firearm suicides in 2018 found that 92% of all firearm deaths in older adults (ages 65^+^) were suicides. In addition, firearms were the most common method (70%) of committing suicide [13]. A study reported by the CDC of adults ages 55 and older found that as males aged their suicide rates increased [14]. Their leading method of suicide was with firearms. However, for older women firearm suicides declined with increasing age as did suicides in general. Finally, a study of firearm injuries in Atlanta, Georgia at one hospital found that 6% (n = 72) of all the firearm injuries from 2016 and 2021 were in older adults (ages 66–74) [15]. The firearm injury intent in these patients was predominantly suicide (95.7%) and 25% of them died from their injuries. Those with suicidal firearm injuries that resulted in death were males (83%), non-Hispanic whites (50%) or non-Hispanic Blacks (39%), and usually lived in distressed communities.

The third form of firearm mortality impacting older adults are unintentional shootings. The U.S. unintentional firearm mortality is four times greater than other high-income countries [16]. Additionally, unintentional firearm deaths are nine times greater in states with the highest level of firearms ownership when compared to states with the lowest availability of firearms [17]. The vast majority of unintentional firearm deaths occur in youths. However, one study on this topic included older adults (60^+^years) [18]. This study found that, assessing 16 states over the years 2005–2015, there were 189 unintentional firearm deaths in which 127 were self-inflicted. The vast majority were males (94.5%) and the most common circumstances were while loading or cleaning the firearm (17.5%), while hunting (17.5%), and when they thought the firearm was not loaded (10.1%). Use of alcohol was suspected in 11.3% of the deaths. One other study of firearm unintentional deaths included individuals divided into 7 age categories, including 55–64, 65–74, and 75 years and older [19]. The cases numbered 84 for the year 1993. Because the number of cases were so few the data were not reported by age groups. However, the study reported the most frequent location of unintentional firearm mortality cases occurred in the house. Also, as the number of guns increased in the house the more likely there would be unintentional firearm deaths.

The purpose of our study was to compare and contrast the three intentions of firearm violence (homicides, suicides and unintentional firearm mortality) in older adults by key demographics. Especially unique in our analysis is years of potential life lost in older adults due to the three forms of firearm violence by key demographics.

## Methods

This study examined firearm mortality in older adults in the United States (U.S.) in a recent typical year, 2021. Older adults were defined as 65 years of age and older. The database for this study was the publicly available data from the U.S. Centers for Disease Control and Prevention (CDC) website, Web-based Inquiry Statistics Query and Reporting System (WISQARS) [1]. WISQARS is a national, regional, and state based mortality database that is a publicly available interactive deidentified database. Because the database we used consists of deceased individuals with their personal information deidentified this study was exempted from Institutional Review Board assessment.

We used descriptive statistics (frequencies, percentages, rank orders and crude rates per 100,000) to characterize the level of fatal firearm violence impacting older adults. Race and ethnicity was delimited to the following groups: non-Hispanic Black, Hispanic and non-Hispanic white. Geographic locations were the CDC identified census regions: Northeast, Midwest, South, and West. The census regions of the CDC has aggregated the states as follows: Northeast (CT, MA, ME, NH, NH, NY, PA, RI, VT), Midwest (IA, IL, IN, KS, MI, MN, MO, ND, NE, OH, SD, WI), South (AL, AR, DC, DE, FL, GA, KY, LA, MD, MS, NC, OK, SC, TN, TX, VA, WV), and West (AK, AZ, CA, CO, HA, ID, MT, NM, NV, DR, UT, WA, WY). The CDC recognizes five categories of intent for firearm mortality: homicides, suicides, unintentional, legal intervention, and undetermined. We delimited our study of older adults to the categories of homicides, suicides, and unintentional firearm mortality.

We computed the potential years of life lost at a lifespan of 80 years (YPLL80) using the CDC standard years of life lost before 80 years for older adults. The ages of firearm trauma deaths of older adults were subtracted from the standard years of life [20, 21]. We assumed virtually every U.S. resident should be able to reach the age of 80 years if they engage in healthy behaviors, have adequate social and financial resources, and have reasonable access to healthcare services [22]. The number of years representing differences between actual ages of death and 80 years of life was summed across all the subjects to obtain the total number of years lost due to firearm injuries.

## Results

During 2021 there were 7645 older adults that died from firearm injuries, approximately 21 everyday, resulting in 45, 980 years of potential life lost before age 80. These figures included firearm deaths as a result of all intents including legal interventions, homicides, suicides, unintentional and undetermined. Firearm deaths of older adults (n = 7580), from the three intents (homicides, suicides, and unintentional) indicates that the vast majority (91.5%) died from suicide inflicted firearm injuries followed by homicide injuries (7.5%). Males were more likely than females to die from all three forms of firearm deaths, as were non-Hispanic whites. Non-Hispanic Blacks died from firearm homicides at a prevalence approximately three times their prevalence in the older adult population (Table 1). In contrast, non-Hispanic whites died from firearm homicides at almost 18% less than their prevalence in the older adult population. The younger age groups (65–69 and 70–74 years) of older adults comprised almost three-fourths (71.8%) of all older adult firearm homicides and the majority (53.6%) of homicides occurred in the South at a rate 40% greater than their prevalence in the older adult population. Males were the majority (61.2%) of firearm homicides, 37% greater than their prevalence in the older adult population.

**Table 1:** Firearm Mortality Demographics of Older Adults, 2021

Topic	Older Adult Pop. (%)	Homicide n	(%)	Suicide n	(%)	Unintentional n	(%)
Total	56,229,126 (100)	575	100	6,935	100	70	100
Gender							
Female	(55.2)	223	(38.8)	593	(8.6)	6*	(8.6)
Male	(44.8)	352	(61.2)	6,342	(91.4)	64	(91.4)
Race/Ethnicity							
Asian (NH)**	(4.8)	11*	(4.2)	37	(1.6)	NA***	
Black (NH)	(9.4)	158	(27.5)	162	(2.3)	NA***	
Hispanic	(9.0)	37	(6.4)	182	(2.6)	NA***	
White (NH)	(75.3)	356	(61.9)	6,486	(93.5)	64	(91.4)
Age (years)							
65–69	(32.4)	244	(42.4)	1720	(24.8)	25	(35.7)
70–74	(27.0)	169	(29.4)	1710	(24.7)	21	(30.0)
75–79	(17.6)	74	(12.9)	1385	(20.0)	10*	(14.3)
80–84	(11.4)	41	(7.1)	1085	(15.6)	10*	(14.3)
85^+^	(11.5)	47	(8.2)	1035	(14.9)	4*	(5.7)
Census region							
Northeast	(18.3)	40	(7.0)	651	(9.5)	NA***	
Midwest	(21.3)	121	(21.0)	1439	(20.7)	14*	(20.0)
South	(37.9)	308	(53.6)	3129	(45.1)	43	(61.4)
West	(22.5)	106	(18.4)	1716	(24.7)	NA***	

* < 20 unstable numbers

**NH non-Hispanic

*** = not available

Source: CDC, WISQARS

In the case of firearm suicides, males (91.4%) non-Hispanic whites (93.5%), and the South (45.1%) had a disproportionate share of these deaths (Table 1). The number of firearm suicides were 12 times the number of firearm homicides and 99 times the number of unintentional firearm deaths. Firearm suicides and homicides declined with age in older adults. However, each age group continued to have over 1000 firearm suicides. Since over 90% of all firearm deaths were due to suicides we explored older adult firearm suicides in states. States with the greatest number of older adult firearm suicides were in the South and Midwest census regions (Table 2). An examination of the states crude rates of most firearm suicides indicate a very different list, with only Arizona appearing on both lists. The majority (7 out of 10) of states on the list of having the highest crude rates were found in the West. In contrast, the 10 states with the fewest number of older adult suicides includes 5 small Northeastern states with smaller populations. The 10 states with the lowest crude rates of firearm suicides have rates approximately one-half the crude rates of the 10 states with the highest rates (Table 2). It should be noted, there are cross-overs in the states (e.g. Alaska, California, Illinois, and Wyoming) where states with large populations of older adults (e.g. California) have high numbers of firearm suicides but low crude rates and states with fewer numbers of older adult firearm suicides (e.g. Wyoming) but have high crude rates of older adult firearm suicides (Table 2).

**Table 2:** Variations in State Suicides in Older Adults, 2021

A. 10 States with Most Suicides and 10 States with Highest Crude Rates			
Number of Suicides		Crude rates* of suicides	
Florida	657	Wyoming	34.45
Texas	558	Montana	28.40
California	524	Nevada	22.99
Arizona	267	Alaska	22.40
Pennsylvania	262	West Virgina	22.30
North Carolina	249	Oklahoma	19.95
Ohio	244	Arizona	19.88
Michigan	235	Arkansas	19.57
Georgia	230	Colorado	19.19
Illinois	193	New Mexico	19.04

B. 10 States with Fewest Suicides and 10 States with Lowest Crude Rates			
Number of Suicides		Crude rates* of suicides	
Rhode Island	< 10**	Massachusetts	3.35
Hawaii	12**	New York	3.97
North Dakota	18**	New Jersey	4.01
Vermont	21	Hawaii	4.18
Alaska	22	Connecticut	8.07
Delaware	22	Minnesota	8.48
South Dakota	23	Nebraska	8.63
Nebraska	28	California	8.73
New Hampshire	31	Illinois	9.12
Maine	36	Maryland	9.57
Wyoming	36		

*Crude rates are per 100,000 older adults

**< 20 unstable number

Source: CDC, WISQARS

Examination of years of potential life lost before age 80 years indicates there were 45,577 years of life lost due to firearm suicides (40, 228), firearm homicides (4,848), and unintentional firearm deaths (501). The vast majority of years lost to firearm deaths occurred in males (67.9–91.6%), non-Hispanic whites (55.8–92.7%), and in the South census region (46.1–63.1%) (Table 3). Because the younger a person dies the more years of potential life is lost, then it should be expected that the youngest age group (65–69 years) lost the most potential years of life (55.6–66.9%). It should be noted, the age groups 80–84 and 85^+^ years are older than the YPLL cut off age of before age 80. Thus, they were not eligible for the YPLL 80 analysis (Table 3).

**Table 3:** Years of Potential Life Lost of Older Adults by Intent of Firearm Mortality, 2021

Topic	Homicide n		(%)	Suicide n		(%)	Unintentional n		(%)
Total	4848	(100)		40,228	(100)		501	(100)	
Gender									
Female	1557	(32.1)		4585	(11.4)		42	(8.4)	
Male	3291	(67.9)		35,643	(88.6)		459	(91.6)	
Race/Ethnicity									
Asian (NH)*	99	(2.0)		257	(0.6)		NA**		
Black (NH)	1554	(32.1)		1076	(2.7)		NA**		
Hispanic	343	(7.1)		1127	(2.8)		NA**		
White (NH)	2703	(55.8)		37,305	(92.7)		452	(90.2)	
Age (years)									
65–69	3242	(66.9)		22,351	(55.6)		310	(61.9)	
70–74	1354	(27.9)		13,655	(33.9)		168	(33.5)	
75–79	262	(5.4)		4222	(10.5)		23	(4.6)	
80–84	NA**			NA**			NA**		
85^+^	NA**			NA**			NA**		
Census Region									
Northeast	310	(6.4)		3555	(8.8)		< 10***	(NA)**	
Midwest	1020	(21.0)		8538	(21.2)		110	(22.0)	
South	2540	(52.4)		18,545	(46.1)		316	(63.1)	
West	978	(20.7)		9590	(23.8)		< 10***	(NA)**	

*NH non-Hispanic

**NA = not available

*** < 20 unstable numbers

Source: CDC, WISQARS

## Discussion

Firearm research regarding U.S. adults have often aggregated all ages without analyses of older age groups. Such aggregation can diminish the public perceptions of the significance of firearm trauma to older adults, especially when the mass media usually reports on the issues of firearm violence of young males. Our study found that males, non-Hispanic whites, those who reside in the South, and younger groups of older adults, were most likely to die from firearm suicides. Whereas males, non-Hispanic Blacks and younger groups of older adults were more likely to be those who died by firearm homicides. Over 9 in 10 firearm deaths in older adults were suicides. The results of previously mentioned firearm studies had results that were for the most part, congruent with our results as it relates to intent, gender, and age (3,14,15). The cost of firearm deaths include the loss of relationships with spouses, aunts/uncles, brothers/sisters, and friends. The firearm deaths also include considerable dollar costs, (e.g. medical, quality adjusted life years, and the value of a statistical life loss). In 2020, it was estimated that in those 65 and older fatal firearm homicides cost $3,080,000,000, firearm suicides cost $30,810,000,000, and firearm unintentional deaths cost $300,000,000 [23]. These dollar costs would be greater if undetermined intent of firearm deaths and non-fatal firearm injuries were included. It is critical that we devise efficacious solutions to the loss of older adults to firearm deaths.

Potential solutions for reducing firearm deaths in older adults include better mental health care, appropriate firearm policies, and improved firearm technology. In the case of mental health care, there are too few specialists. It is considered not “normal” to want to take your life. In 2019, it was estimated there were 660 geropsychologists (1% of all doctoral level psychologists) and at the end of 2022 there were 1,354 board certified geriatric psychiatrists in the United States [24, 25]. In addition, there are problems with geographic access, distribution is concentrated in urban populations. Psychiatry is the medical specialty least likely to accept health insurance and the majority of geriatric psychiatrists are international medical graduates (IMGs) which may create distrust in some older adults [26]. In addition, older adults are known to have psychiatric disorders, mood disorders (6.8%), substance use disorders (3.8%), anxiety disorders (11.4%), and personality disorders (14.5%) and most mental health disorders decrease with increasing age. Some of these disorders are risk factors for suicide [27]. Noteworthy is that firearm homicides and suicides also decrease with age in older adults. Another issue with receiving mental health care is that Medicare limits the number of days, 190 days per lifetime for inpatient mental health care. This is the only health issue that is lifetime limited by Medicare in the number of hospital inpatient days [28]. For decades, we have been failing the mental health needs of our citizens.

The second area for mitigating some forms of firearm mortality is through state and federal legislation. Extreme risk protective orders (ERPOs) also known as “red flag laws”, exist in 21 states and the District of Columbia as of December 2024. These laws allow a variety of people (e.g. law enforcement, family members, physicians, educators, depending on the state) to petition a court to temporarily restrict, usually not more than a year, a person's access to firearms by a civil restraining order [29]. The state representative must prove to the court that the person is an imminent threat of harming themselves or others. Law enforcement officers remove firearms owned by the subject and prohibit the purchase of more firearms during the time of the restraining order. Two early studies in Connecticut and Indiana estimated that for every 10 to 20 ERPO actions taken, one firearm suicide was averted [30, 31]. A more recent study of ERPOs in four states (CA, CT, MD and WA) found that in those who expressed suicide intent, it took 13 to 18 cases to avert one suicide [32]. The study also found when firearms were returned to their owners a number of owners went on to commit firearm suicides. As the authors noted, simply removing firearms for a period of time does not address the underlying risk factors that push individuals to believe they would be better off dead. We encourage states without ERPOs to consider adopting them.

Child access prevention (CAP) laws, also known as safe storage laws, reduce unintentional firearm injuries among adults [29]. States without negligent storage CAP laws should adopt them or a similar law to help reduce unintentional firearm deaths in older adults. Since 1998, the National Instant Criminal Background Check System (NICS) has required NICS background checks for all firearm purchases from federally licensed firearm dealers. The FBI typically has three days to investigate whether the purchaser has been reported to be an ineligible purchaser. The federally licensed firearm dealer has the right after three days to transfer the firearm to the purchaser. Most (90%) FBI reviews can be completed in minutes or hours. This law may be helpful for older adults who are in a crisis and intend to impulsively commit suicide [33, 34]. However, this law would only be effective for older adults who are purchasing a firearm. In addition, research has shown that handgun purchases had an elevated risk of firearm suicides during the month after they purchased their handgun [35]. A large portion of the older adults likely purchased their firearm when they were younger. The underlying risk factors pushing an older adult to commit suicide will likely still be impacting the individual after their firearm is transferred to them. We believe states without waiting periods should adopt them and they should be longer than the NICS waiting timeframe.

The third area that might reduce firearm related deaths in older adults is personalized firearm technology. The most common method of using this technology is with personalized firearms also known as “smart guns” [36]. This firearm technology ensures that a firearm can be fired only by an “authorized” person. The technology uses radio frequency identification, (RFID) or a biometric identification (e.g. fingerprint) to identify the authorized user [37]. Another use of this technology could be with firearm storage boxes. Since firearms, especially handguns, are purchased for protection, and many of those owners want quick availability to their loaded firearm, then a storage box with smart technology might be more acceptable than a comparable firearm. This technology could help reduce unintentional firearm deaths, and if the older adult did not own their own firearm but tried to access a firearm owned by a person they lived with, they would not be able to use it because they were not authorized. A major obstacle to adoption of this technology may be the high cost of this technology [37]. The federal government may need to explore the issue of subsidizing the purchase of such technology to help reduce firearm mortality. The last issue we would like to draw attention to would be for semi-automatic handguns to be required to have loaded chamber indicators to let the owner know a round is in the chamber. This firearm indicator could help reduce unintended firearm deaths in older adults. Unfortunately, empirical evidence on the effectiveness of this new technology is not available.

Our study has several potential limitations [38]. The WISQARS database did not contain numerous demographic variables such as education level, economic statuses, place of death, marital status and other variables (e.g. type of firearm, substance abuse, etc.) that could have assisted with a more granular assessment of older adult firearm deaths. Our findings may be limited by inaccuracies in codings of the intent of firearm fatalities [39]. We conducted our analysis of YPLL, based on a projected lifespan to 80 years of age. However, actuarial tables could be used to estimate potential years of life lost to firearm fatalities by those older than 80 years of age. Thus, our YPLL analysis underestimates the number of total years lost by older adults to firearm fatalities. Our study strengths lies in being the first study to attempt an assessment of YPLL due to firearm mortality in older adults. Additionally, our comparison of the three leading forms of firearm mortality in older adults can help guide preventive policy efforts to eliminate firearm trauma in older adults.

## Conclusion

Firearm deaths in older adults are primarily firearm suicides. Firearms are the most common means used in older adult homicides and suicides. Focusing preventive efforts primarily on the instrument (e.g. firearms) of firearm deaths will not likely make a major reduction in such deaths, especially firearm suicides. A major focus on the underlying risk factors for firearm trauma in older adults is an essential component of preventing firearm trauma.
